# Analysis of cartilage injury patterns and risk factors for knee joint damage in patients with primary lateral patella dislocations

**DOI:** 10.1371/journal.pone.0258240

**Published:** 2021-10-14

**Authors:** Yannick Palmowski, Tobias Jung, Anne-Katrin Doering, Clemens Gwinner, Imke Schatka, Benjamin Bartek

**Affiliations:** 1 Center for Musculoskeletal Surgery, Charité –Universitätsmedizin Berlin, Corporate Member of Freie Universität Berlin, Humboldt-Universität zu Berlin, and Berlin Institute of Health, Berlin, Germany; 2 Center for Diagnostic and Interventional Radiology and Nuclear Medicine, Charité –Universitätsmedizin Berlin, Corporate Member of Freie Universität Berlin, Humboldt-Universität zu Berlin, and Berlin Institute of Health, Berlin, Germany; Paracelsus Medizinische Privatuniversitat - Nurnberg, GERMANY

## Abstract

**Background:**

Lateral patellar dislocation (LPD) frequently causes damage to the knee. Injury patterns and risk factors contributing to such injuries have not yet been examined in detail.

**Methods:**

We retrospectively analyzed 50 consecutive patients with primary LPD. Two reviewers evaluated the MRI images regarding risk factors for LPD (Dejours classification; Caton-Deschamps Index, CDI; distance from the tibial tuberosity to trochlear groove, TT-TG; trochlear depth, TD) as well as joint damages according to the Whole-Organ Magnetic Resonance Imaging Score (WORMS).

**Results:**

33 male and 17 female patients with a mean age of 23.2 (±9.6) years were included in this study. 52% were classified Dejours ≥ B, 34% had a CDI ≥ 1.3, 22% a TT-TG ≥ 20mm and 52% a TD < 3mm. 49 out of 50 patients (98%) showed abnormalities according to WORMS. The most frequently observed abnormalities were synovitis/effusion (49/50, 98%), bone marrow oedema (44/50, 88%) and cartilage damage (42/50, 84%). Most frequently affected subregions were medial (41/50, 82%) and lateral (31/50, 62%) patella as well as the anterior (43/50, 86%), central (42/50, 84%) and posterior (11/50, 22%) portion of the lateral femoral condyle. There was no significant correlation between any of the examined risk factors and joint damages according to WORMS. Male patients had higher scores regarding total cartilage damage (5.11 vs. 2.56, p = 0.029), total score for the lateral femorotibial joint (3.15 vs. 1.65, p = 0.026) and overall total WORMS score (12.15 vs. 8.29, p = 0.038).

**Conclusion:**

Risk factors for LPD do not influence the risk of damages to the knee joint after primary LPD. Although LPD is generally known to affect more female than male patients, male patients suffered more severe injuries after primary LPD, particularly of the lateral femorotibial joint. Overall, our results underline the importance of MRI imaging after primary LPD.

## Introduction

First time lateral patellar dislocation (LPD) is a common injury in young, physically active patients with an annual incidence of 23.2 per 100,000 person-years [1]. It predominantly affects female patients and has the highest incidence among adolescents aged 14 to 18 years (147.7/100,000 person-years) [1–4]. In addition to a thorough clinical examination, MRI are usually performed to confirm the diagnosis and to evaluate potential damage to the knee joint. Contusion of the lateral femoral condyle and the medial patella border have been reported in up to 100% of all patients after primary LPD and confirm the diagnosis [5, 6]. Previous studies have demonstrated that chondral and osteochondral injury are common in patients with patellar instability [7–9]. Concomitant cartilage lesions are reported in up to 97% of patients after LPD [10, 11]. As such cartilage lesions have been shown to deteriorate over time, leading to osteoarthritic changes even in compartments other than the patellofemoral joint (PFJ) in the knee, they may cause serious long-term sequelae for affected patients [3].

Apart from the evaluation of knee joint damage, MRI also provides important information regarding the joint geometry and known risk factors for lateral patellar instability such as increased tibial-tuberosity to trochlear groove distance (TT-TG), patella alta, and trochlear dysplasia [12–14]. Despite the considerable amount of literature regarding the risk factors of LPD and its epidemiology, only few studies have examined the exact injury patterns and the risk factors for severe knee joint damage after LPD and did not come to consistent conclusions. While Farr at al. state that primary LPD in patients with “normal” patellofemoral anatomy leads to more severe chondral damage than patella dislocations in patients with patellofemoral dysplasia and/or patella alta due to the higher amount of energy required, Tompkins et al. reported that underlying anatomic risk factors for PF instability do not predict injury patterns [6, 15].

The aim of the present study was to describe the injury patterns of chondral lesions after primary lateral patellar dislocation (LPD) in detail using the most accurate and detailed Whole-Organ Magnetic Resonance Imaging Score (WORMS) for the knee joint and to identify risk factors for resulting knee joint damages [16].

## Materials and methods

### Patients

50 consecutive patients were included that presented with a primary lateral dislocation of the patella in the casualty department or the outpatient clinic of our maximum-care university hospital and that received magnetic resonance imaging (MRI) scans of the affected knee between February 2007 and September 2012 at our institution. Inclusion criteria were the history of a primary lateral dislocation of the patella and the availability of MRI images after the luxation. Exclusion criteria were recurrent patella dislocations, insufficient or incomplete images and dislocations due to a direct trauma to the patella.

### Radiographic evaluation

MRIs were performed with a 2D coronal proton-density (PD) weighted turbo spin-echo sequence with fast suppression using the following parameters: 0.4167 × 0.4167 mm in-plane resolution, 3 mm slice thickness, 3.6 mm slice spacing, 29 ms echo time, 3520 ms repetition time, 150° flip angle. In addition, a 3D axial T2-weighted Multi-Echo Data Image Combination (MEDIC) sequence was performed with 0.167 × 0.167 mm in-plane resolution, 1.2 mm slice thick-ness, 21 ms echo time, 38 ms repetition time and 8° flip angle. All patients were positioned in supine position with the leg in full extension. Two reviewers, both specialized knee surgeons, evaluated the MRI images regarding damages to the knee joint and risk factors for patella dislocation. Examined risk factors included the configuration of the trochlea according to the Dejours classification, the Caton-Deschamps Index (CDI) for patellar height, the distance from the tibial tuberosity to trochlear groove (TT-TG) and the trochlear depth (TD) [17]. Abnormal values were defined as > 1.3 for CDI, > 20mm for TT-TG and < 3mm for TD. Trochlea morphology was assessed on the first proximal transverse MRI plane that showed a completely cartilage-covered trochlea. The trochlear depth was assessed by measuring the distances between the posterior condyle line and the medial (MCD) and lateral condyles (LCD) as well as the lowest part of the trochlear groove (LTG). Trochlear depth was calculated according to the formula (MCD + LCD) / 2-LTG [18]. The morphological classification is based on the four types described by Dejour et al. [19]. In type A, the trochlear preserves its concave shape but has shallow trochlear groove; type B is flattened or convex trochlea; in type C, the medial facet is hypoplastic (facet asymmetry) with high lateral facet, resulting in flattened joint surface in an oblique plane; and type D shows a “cliff pattern” with type C features and a vertical link between the medial and lateral facets.

Injuries of the knee joint were evaluated using the Whole-Organ Magnetic Resonance Imaging Score (WORMS). The WORMS is a highly elaborated semi-quantitative scoring method for the whole-organ evaluation of the knee joint based on MRI [16]. It measures 14 different features (articular cartilage integrity, subarticular bone marrow abnormality, subarticular cysts, subarticular bone attrition, marginal osteophytes, medial and lateral meniscal integrity, anterior and posterior cruciate ligament integrity, medial and lateral collateral ligament integrity, synovitis/effusion, intraarticular loose bodies, and periarticular cysts/bursitis) in 4 regions (patellofemoral joint, medial femorotibial joint, lateral femorotibial joint, subspinous region) and 15 subdivisions (e.g. anterior/central/posterior region of the lateral femoral condyle) of the knee. For each feature, a separate score is calculated for each region and each subdivision as well as a total score. High scores represent relevant knee joint damage whereas a score of 0 would mean the absence of radiological evidence of knee joint damage. Thus, the WORMS offers a very detailed assessment of the knee joint, and also has a high inter-rater reliability [16].

### Statistical analysis

Statistical analysis was performed using SPSS software version 27 (IBM, New York, USA). Mean and standard deviation were calculated for descriptive patient characteristics. Correlations were assessed using Spearman’s rank correlation coefficient. Means between groups were compared using Student’s T-Test for parametric parameters (for the variables age, CD, TT-TG and TD) or Mann-Whitney-U-Test for non-parametric variables (for WORMS Scores), as appropriate. Chi-Square test was used to compare the frequency of risk factors for LPD between male and female patients. The level of significance was defined as p < 0.05.

### Ethics approval

The study was approved by the institutional review board of Charité –Universitätsmedizin Berlin (EA1/374/20).

## Results

### Study population

A total of 50 patients (33 m, 17 w) with an average age 23.2 years (±9.6, 11–50) were included in the study. 6 patients (12%) had open physis. Abnormal values for the risk factors were found in 17 patients (34%) for CDI, 11 patients (22%) for TT-TG and 26 patients (52%) for TD. 7 patients (14%) had severe trochlea dysplasia of Dejours type C or D, and 4 of these 7 patients (57.1%) also had a pathological TT-TG of > 20mm. A summary of the patient characteristics is shown in **Table 1**.

**Table 1:** Patient characteristics.

Patients (n)	50
Male	33 (66%)
Female	17 (34%)
Age (years; SD, range)	23.2 (±9.6)
Dejours classification	
A	24 (48%)
B	19 (38%)
C	5 (10%)
D	2 (4%)
CDI (SD, range)	1.2 (± 0.2, 0.9–1.7)
CDI > 1.3 (n)	17 (34%)
TT-TG (SD, range)	15.1 (± 5.9, 0–28)
TT-TG > 20mm (n)	11 (22%)
TD (SD, range)	2.8 (±1.4, -0.7–5.6)
TD < 3mm (n)	26 (52%)

CDI: Caton-Deschamps Index, TT-TG: distance from the tibial tuberosity to trochlear groove, TD: trochlear depth, SD: standard deviation.

### Injury patterns

Overall, 98% of the patients showed abnormal scores according to WORMS. The most frequent finding was synovitis/effusion, which occurred in 98%, followed by bone marrow abnormalities (88%) and cartilage damage (84%). Regarding the region of the injuries, the patellofemoral joint (PFJ, 94%) and the lateral femorotibial joint (LFTJ,84%) were most frequently affected. An overview of the localization of the observed abnormalities is presented in **Table 2**. The highest average score was also observed for the PFJ (5.96 ±.3.84, 0–16), followed by the LFTJ (2.64 ±.2.45, 0–11) (**Table 3**).

**Table 2:** Number of patients with abnormalities of the knee joint according to WORMS.

Feature	MFTJ	LFTJ	PFJ	S Region	Total
Cartilage	1 (2%)	20 (40%)	41 (82%)	-	42 (84%)
Marrow abnormality	2 (2%)	39 (78%)	44 (88%)	0	44 (88%)
Bone cysts	1 (2%)	2 (4%)	0	0	3 (6%)
Bone attrition	0	0	0	-	0
Osteophytes	1 (2%)	0	0	-	1 (2%)
Compartment total	4 (8%)	42 (84%)	47 (94%)	-	-
Menisci	1 (2%)	0	-	-	1 (2%)
Ligaments	-	-	-	-	5 (10%)
Synovitis/effusion	-	-	-	-	49 (98%)
Loose bodies	-	-	-	-	7 (14%)
Periarticular cysts/bursities	-	-	-	-	5 (10%)
Total	4 (8%)	42 (84%)	47 (94%)	0	49 (98%)

MFTJ: medial femorotibial joint, LFTJ: lateral femorotibial joint, PFJ: patellofemoral joint, S Region: subspinous region.

**Table 3:** Average WORMS of separate regions and in total.

Feature	MFTJ (±SD, range)	LFTJ (±SD, range)	PFJ (±SD, range)	S Region (±SD, range)	Total (±SD, range)
Cartilage	0.02 (±0.14, 0–1)	0.90 (±1.54, 0–6)	3.32 (±2.99, 0–11.5)	-	4.24 (±3.63, 0–15)
Marrow abnormality	0.02 (±0.14, 0–1)	1.70 (±1.45, 0–5)	2.64 (±1.56, 0–6)	0.00 (±0.00, 0–0)	4.36 (±2.69, 0–11)
Bone cysts	0.02 (±0.14, 0–1)	0.04 (±0.20, 0–1)	0.00 (±0.00, 0–0)	0.00 (±0.00, 0–0)	0.06 (±0.24, 0–1)
Bone attrition	0.00 (±0.00, 0–0)	0.00 (±0.00, 0–0)	0.00 (±0.00, - 0)	-	0.00 (±0.00, - 0)
Osteophytes	0.02 (±.14, 0–1)	0.00 (±0.00, - 0)	0.00 (±0.00, - 0)	-	0.02 (±0.14, 0–1)
Menisci	0.08 (±0.57, 0–4)	0.00 (±0.00, - 0)	-	-	0.08 (±0.57, 0–4)
Ligaments	-	-	-	-	0.10 (±0.30, 0–1)
Synovitis/effusion	-	-	-	-	1.74 (±0.80, 0–3)
Loose bodies	-	-	-	-	0.16 (±0.42, 0–2)
Periarticular cysts/bursities	-	-	-	-	0.10 (±0.30)
Total	0.08 (±0.27, 0–1)	2.64 (±2.45, 0–11)	5.96 (±3.84, 0–16)	0.00 (±0.00, 0–0)	10.84 (±6.12, 1–28)

MFTJ: medial femorotibial joint, LFTJ: lateral femorotibial joint, PFJ: patellofemoral joint, S Region: subspinous region.

### Risk factors for knee joint damage

**Fig 1:**
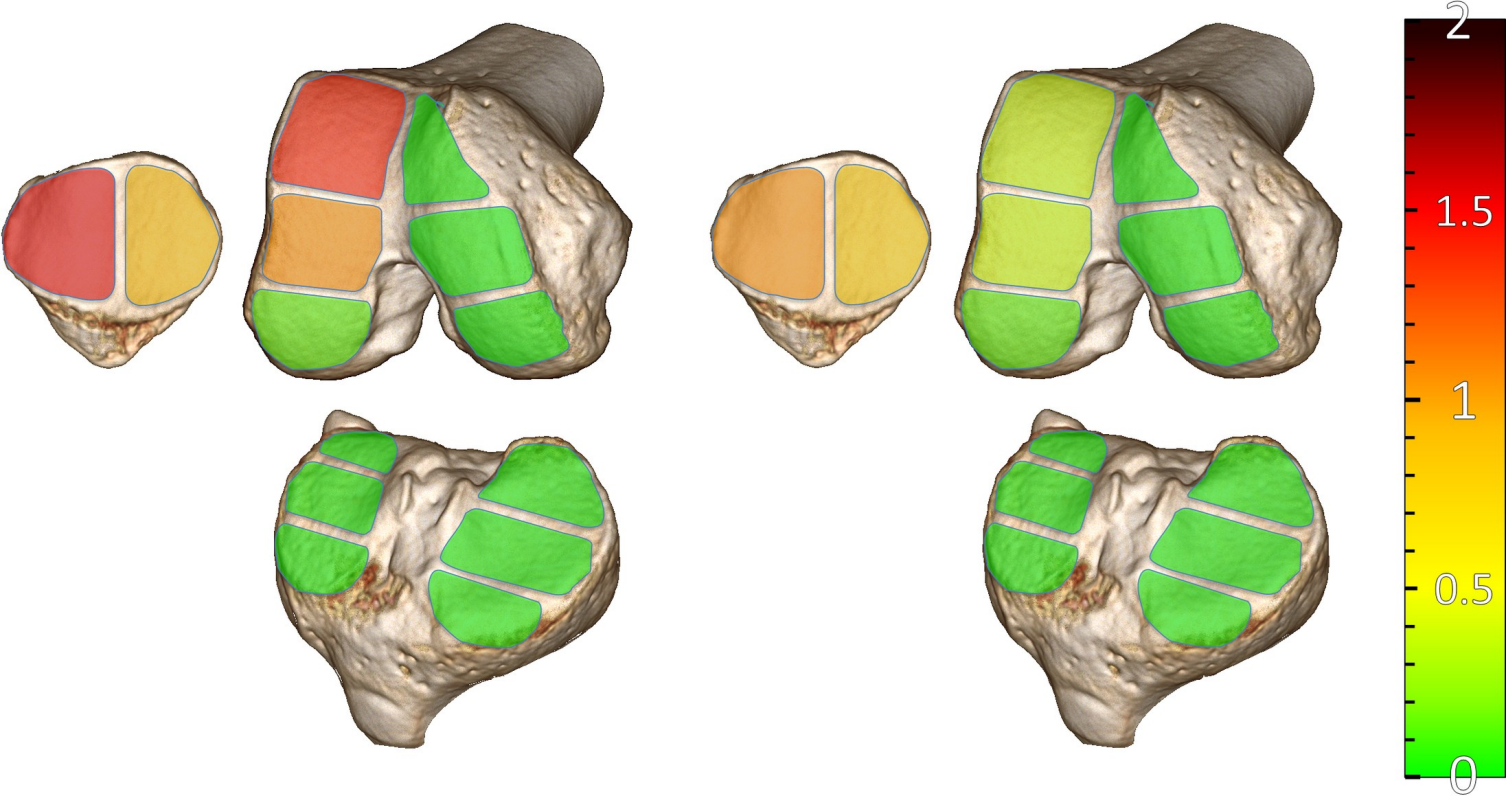
Average cartilage damage of the various subregions in male (left) and female (right) patients according to WORMS.

The frequency of anatomical risk factors for LPD did not significantly differ between male and female patients (TT-TG > 20mm: 24.2% vs. 17.6%, p = 0.594; CD > 1.3: 33.3% vs. 35.3, p = 0.89; TD < 3mm: 48.5% vs. 58.8, p = 0.488). WORMS of the MFTJ, LFTJ, PFJ and total scores did not significantly differ between patients with normal or abnormal CDI, TT-TG and TD (**Table 4**) and there was no significant correlation between any of these parameters and WORMS of the MFTJ, LFTJ, PFJ or total scores. Additionally, there was no significant correlation between these anatomical parameters and cartilage damage of separate subregions. The comparison between male and female patients showed significant higher scores for males regarding the total cartilage damage (5.11 vs. 2.56, p = 0.029) as well as the overall total score (12.15 vs. 8.29, p = 0.038) **(Table 5)**. Regarding the region of joint damage, particularly the LFTJ was more frequently affected in male patients with significant differences in total WORMS (3.15 vs. 1.65, p = 0.026). A comparison of the severity of cartilage damages in male and female patients is depicted in **Fig 1**.

**Table 4:** Influence of anatomical risk factors for patella dislocation on knee joint damage.

Feature	CDI		TT-TG		TD	
	Z	p	Z	p	Z	p
MFTJ Total	-0.39	0.695	-1.40	0.163	-0.08	0.934
LFTJ Total	-0.41	0.685	-0.50	0.618	-0.62	0.535
PFJ Total	-0.26	0.797	-0.835	0.404	-1.37	0.172
Total Score	-0.19	0.85	-0.29	0.77	-1.1	0.27

**Table 5:** Comparison of WORMS scores between male and female patients.

Feature	Male		Female		p-value
	Mean	SD (range)	Mean	SD (range)	
Age	24.91	10.26 (14–50)	19.94	7.46 (11–38)	0.084
Caton Deschamps Index	1.2	0.2 (0.9–1.67)	1.2	0.2 (1–1.7)	0.964
TT-TG	15.4	6.2 (0–28)	14.3	5.4 (0–22)	0.528
Trochlear Depth	2.9	1.3 (0–5.15)	2.6	1.8 (-0.65–5.55)	0.599
Cartilage Total	5.11	3.97 (0–15)	2.56	2.05 (0–6)	0.029
Marrow Abnormality Total	4.79	2.38 (0–9)	3.53	3.10 (0–11)	0.078
MFTJ Total	0.09	0.29 (0–1)	0.06	0.24 (0–1)	0.695
LFTJ Total	3.15	2.61 (0–11)	1.65	1.77 (0–6)	0.026
PFJ Total	6.71	4.24 (0–16)	4.5	2.41 (0–10)	0.082
Total Score	12.15	6.4 (2–28)	8.29	4.73 (1–20)	0.038

SD: Standard Deviation, MFTJ: medial femorotibial joint, LFTJ: lateral femorotibial joint, PFJ: patellofemoral joint.

## Discussion

The present study aimed to analyze patterns of knee injury in patients after primary patellar dislocations and elucidated potential risk factors. While the majority of published MRI studies on patellar dislocations include a mixed population of patients with primary and recurrent patellar dislocations, this study provides data on MRI findings in a cohort of patients exclusively with primary LPD [20–23]. For this purpose, multiple widely used risk factors of patellar dislocations were assessed on MRI images and knee joint damage was evaluated according to the WORMS, an elaborate state-of-the-art tool allowing a highly detailed analysis of type and localization of damages.

In our study, almost all patients (98%) had elevated WORMS scores after primary patellar dislocation. This confirms results of previous studies, which also report knee joint damages in up to 100% of the affected patients [5, 6, 10, 11]. Apart from joint effusion, the most frequently occurring abnormalities were bone marrow oedema (88%) and cartilage damage (84%). While cartilage damage can cause long-term sequelae and accelerate the occurrence of osteoarthritis, bone marrow oedema can be considered as a footprint of the mechanism of injury [3, 24]. The even higher number of patients with bone marrow oedema compared to cartilage damage demonstrates the considerable forces acting on the knee joint during primary patella dislocation also in patient with no evident resulting cartilage damage. Regarding the localization of injuries, the most affected regions were the patellofemoral joint and the lateral tibiofemoral joint. In specific, the most frequently affected subregions were the medial (41/50, 84%) and lateral (31/50, 62%) patella as well as the anterior (43/50, 86%) and central (42/50, 84%) portion of the lateral femoral condyle. The high concentration of damages in these regions together with the paucity of lesions in the medial compartment make it highly likely that the injuries were actually caused by the dislocation and no incidental pre-existing findings. Overall, these high numbers underline the severity of even singular patellar dislocation and the importance of MRI imaging in any affected patient.

Hardly any patients showed abnormal WORMS scores in the categories bone attrition (0/50) or osteophytes (1/50). This was to be expected in our cohort, as the included patients were predominantly young (mean age 23.2 years) and these categories are more important for the evaluation of osteoarthritis. The only patient with osteophytes had a single point in one single subregion (anterior region of the medial tibial surface) and was considerably older than the average (36 years).

A large portion of the included patients showed anatomical risk factors for patella dislocations. Only 17/50 patients (34%) did not show abnormal values of CDI, TT-TG or TD and 26/50 patients (52%) had a trochlea dysplasia type B, C or D according to the Dejours classification. These numbers are overall in line with those from previous studies. One other existing study also focusing on patients with primary patellar dislocation reported almost identical numbers for CDI (1.24 vs. 1.23), TT-TG (15.06 vs. 15.1) and TD (2.78 vs. 2.7) [25]. This confirms the high clinical relevance of these parameters for the evaluation of the risk for patellar dislocation.

However, the presence of these risk factors only seems to increase the risk of LPD, but not the risk of suffering damage to the knee joint in case of LPD. There was no correlation between any of the examined risk factors (CDI, TT-TG, TD) and there was no difference in WORMS between patients with normal and abnormal values of these anatomical risk factors. Similar observations were made by a previous study on primary LPD [15]. Stratification by gender revealed male sex to be associated with higher scores for cartilage damage and also higher total WORMS scores. This might seem surprising, since women are generally known to have a higher incidence of LPD than men [4]. However, the higher risk of LPD in women and the higher risk of knee joint damage in men might actually have the same reason: the overall higher laxity of female knees reduces the necessary forces to cause a dislocation of the patella and could thereby also reduce the average acting forces during LPD [26]. This is also a probable reason for the similarly unexpected gender distribution in our study, where two thirds of the patients were male. Patients were included from the casualty department or the outpatient clinic of our maximum-care university hospital, where patients usually only present in case of relevant subjective discomfort. It can be presumed that some women do not see a doctor after LPD or wait for an appointment with their ambulant orthopedic doctor due to lack of severe pain. Similar observations were made in a previous study, which only included patients with bone bruise in MRI and in which around two thirds of the participants were male as well (127 male, 68 female) [27].

Strengths of our study include strict inclusion criteria only considering patients with primary LPD in contrast to the vast majority of existing studies that examine mixed cohorts of primary and recurrent LPD. Additionally, a systematic in-depth analysis of all MRIs was performed using the WORMS as a state-of-the-art tool for the assessment of knee joint damage. Still, there are limitations to our study. The present study is retrospective with the inherent limitations. For this reason, no a priori power analysis was performed, which limits the conclusions that can be drawn. For example, it is possible that correlations between anatomic risk factors for LPD and knee joint damage according to WORMS were not detected due an insufficient number of participants. Post hoc sensitivity analysis shows that our study was powered to detect correlations of R ≥ 0.375 with a power of at least 0.8. As the exact time of the dislocations was not recorded, no clear differentiation between acute and chronic changes was possible. Although no radiologists were involved, the radiographic measurements were performed by specialized knee surgeons experienced with the radiographic evaluation of MRI studies of the knee. Additional factors that might possibly influence knee joint damages after LPD could be varus or valgus malalignments. Unfortunately, respective data were not available for all patients so that no analysis regarding their influence was possible. Since some patients continued their treatment externally, it was not possible to analyze the number of cases in which cartilage addressing procedures like flake refixation or cartilage repair was necessary. As elaborated above, more female than male patients were included, although LPD is known to generally affect more women than men. This is most likely due to a selection bias, as only patients with strong enough discomfort to present in hospital were included. As it can consequently be presumed that more female than male patients only have light symptoms after primary LPD, the gender differences observed in our study might actually even be higher. Furthermore, no follow-up was conducted so that no definite conclusions regarding the long-term sequelae can be drawn.

## Conclusion

Our results suggest that the presence of risk factors for LPD does not significantly alter the risk of damages to the knee joint after primary LPD. Although LPD is generally known to affect more female than male patients, male patients seem to suffer more severe injuries after primary LPD, particularly of the lateral femorotibial joint. Overall, our results underline the importance of acute MRI imaging after primary LPD.
